# Pan-cancer clinical impact of latent drivers from double mutations

**DOI:** 10.1038/s42003-023-04519-5

**Published:** 2023-02-20

**Authors:** Bengi Ruken Yavuz, Chung-Jung Tsai, Ruth Nussinov, Nurcan Tuncbag

**Affiliations:** 1 Graduate School of Informatics, Department of Health Informatics, Middle East Technical University, Ankara, 06800 Turkey; 2 Computational Structural Biology Section, Frederick National Laboratory for Cancer Research, National Cancer Institute at Frederick, Frederick, MD 21702 USA; 3 Department of Human Molecular Genetics and Biochemistry, Sackler School of Medicine, Tel Aviv University, Tel Aviv, 69978 Israel; 4 Department of Chemical and Biological Engineering, College of Engineering, Koç University, Istanbul, 34450 Turkey; 5 School of Medicine, Koç University, Istanbul, 34450 Turkey; 6 Koc University Research Center for Translational Medicine (KUTTAM), Koç University, Istanbul, 34450 Turkey

**Keywords:** Computational biology and bioinformatics, Cancer

## Abstract

Here, we discover potential ‘latent driver’ mutations in cancer genomes. Latent drivers have low frequencies and minor observable translational potential. As such, to date they have escaped identification. Their discovery is important, since when paired in *cis*, latent driver mutations can drive cancer. Our comprehensive statistical analysis of the pan-cancer mutation profiles of ~60,000 tumor sequences from the TCGA and AACR-GENIE cohorts identifies significantly co-occurring potential latent drivers. We observe 155 same gene double mutations of which 140 individual components are cataloged as latent drivers. Evaluation of cell lines and patient-derived xenograft response data to drug treatment indicate that in certain genes double mutations may have a prominent role in increasing oncogenic activity, hence obtaining a better drug response, as in *PIK3CA*. Taken together, our comprehensive analyses indicate that same-gene double mutations are exceedingly rare phenomena but are a signature for some cancer types, e.g., breast, and lung cancers. The relative rarity of doublets can be explained by the likelihood of strong signals resulting in oncogene-induced senescence, and by doublets consisting of non-identical single residue components populating the background mutational load, thus not identified.

## Abstract (web summary)

A statistical methodology is used to identify potential ‘latent driver’ mutations from public cancer bulk sequencing data.

## Introduction

Cancer is a disease of uncontrolled cell proliferation driven by molecular alterations. The impact of these alterations diffuses into the molecular interaction network and changes signaling pathways and transcriptional regulation in the cell. Not all alterations equally contribute to a growth advantage of cancer cells. Some mutations are drivers; others are passengers^1^. Whereas it is generally believed that passenger mutations do not bestow proliferative effects on the disease phenotype, their properties, and possible roles are not fully understood^2^. Cancer genomics and evolution studies suggest that the accumulation of ‘slightly’ deleterious passenger mutations can slow cancer progression, and this could be exploited for therapeutic purposes^3^. Lately, another class of mutations was defined, dubbed “latent” or “mini-drivers”^4–6^. Even though not identified as drivers since the effect that latent drivers generate is marginal, when coupled with other activating mutations, latent mutations can additively intensify the signal. Their detection may help forecast cancer progression and improve personalized treatment strategies^5^. Curated driver genes and mutations have been deposited in multiple databases^7–9^ and used to develop computational approaches to predict driver genes and driver mutations^10–15^. These methods, including frequency-based methods, subnetwork identification methods, and 3D mutation search methods, have been comprehensively compared^16–20^. One of the concerns with frequency-based approaches is that prohibitively large sample sizes are needed to identify infrequently mutated driver genes. Thus, in frequency-based approaches, there is a risk of generating biased results due to background mutation rates^21,22^. There are various resources and web servers that examine the effect of missense mutations on protein stability, protein–protein interactions, and the underlying molecular mechanisms^23,24^. However, frequency-based approaches fail in the identification of rare drivers which can be tissue-specific^25^. A recent multidimensional analysis of cancer driver genes in IntOGen showed that some drivers are cancer-wide whereas others are specific to a limited number of cancer types^12^.

Even a single mutation in a gene can be considered as a prognostic marker and change the global genome and protein expression, eventually altering the signaling pathways^26^. However, it has been estimated that the contribution of a single driver mutation to cancer progression is very small and needs additional mutations over time^27^. Despite DNA repair, somatic mutations accumulate and different genotypes in individual tissues are generated. This mechanism, called ‘somatic mosaicism’, offers driver, or synergistic mutations an advantage in cancer cells^28^. Recently, the combination of single frequent mutations with a rare, or weak mutation in the *same* gene was shown to have a substantial advantage in tumor progression and influence treatment response. These double mutations *in cis* in *PIK3CA* were shown to be more oncogenic, and more sensitive to inhibitors compared to a single mutation^29^. A recent work cataloged ‘composite mutations’ of *multiple* genes having more than one non-synonymous mutation in the same tumor^30^. Saito et al. demonstrated the functional implications of multiple driver mutations in the same oncogene with an emphasis on *PIK3CA*^31,32^.

Here, aided by informatics techniques, we systematically screen somatic mutations in pan-cancer data across ~60,000 patient tumors. We aim to find co-occurring patterns that are predominantly present in specific tissues and tumor types. Our screening reveals tumor-type specific double mutations in the same gene which may promote tumorigenesis and alter the response to treatments. It also reveals that tumors having at least one double mutation pair may lead to changes in response to drugs. We cataloged the components of double mutations as latent mutations if their co-occurrence is statistically significant and not yet labeled as a cancer driver. This led us to uncover 140 latent driver mutations. The oncogenic activation of the protein may be through a single, or multiple additive contributions of the mutations. Although the existence of a set of driver genes is considered cancer-wide, we show that having double mutations in those genes is cancer-specific. Same gene double mutations are relatively rare; however, their impact is elevated in tumor progression.

## Results

### Discovery of latent drivers through double mutations

Multiple mutations in a single gene rarely co-occur in patient tumors. Vasan et al. examined the *PIK3CA*-mutant cancer genomes and reported that 12–15% of breast cancers and other tumor types harbor multiple *PIK3CA* mutations, the majority of which (95%) are double mutations^29^. Similarly, Saito et al. performed a pan-cancer study to check the presence of multiple mutations in a subset of oncogenes among ~60,000 tumors. They discovered 20 oncogenes with a higher rate of multiple mutations than expected where 9% of samples with at least one mutation in these oncogenes had multiple mutations^31,32^. Despite their relative scarcity, when multiple mutations are together in the same gene, they may cause dramatic phenotypic differences and can be signatures of specific tumor tissues or cancer types^29–31^. For example, double mutations in *PIK3CA* increase the sensitivity to PI3K inhibitors in breast cancer^29^, while double mutations in *EGFR* predominantly exist in lung cancer^33^. We defined latent driver mutations as mutations that have not been associated with tumor development due to their unobservable translational or structural effects. However, when combined with other alterations, can contribute to cancer progression and drug resistance^5^. Some mutations cataloged as passengers may belong to this category. The collective action of latent driver mutations in oncogenes (OGs) can switch the protein ensemble to an active state; in tumor suppressor genes (TSGs) the inactive state. When the mutations are on the same allele (i.e., *in cis*), a latent driver mutation could couple with driver mutations; two or more latent driver mutations can also collaborate. In either case, the outcome can have a stronger effect. Along similar lines, a strong driver may couple with a weak driver or a latent driver, strengthening the pathological impact. Our definition of latent mutations applies only to mutations *in cis*. That is, in the same protein molecule (i.e., multiple same-allele driver mutations). Allosteric effects cannot be applied in trans, that is, to mutations in two different molecules, where one molecule has one mutation and the other has the second.

**Fig. 1:**
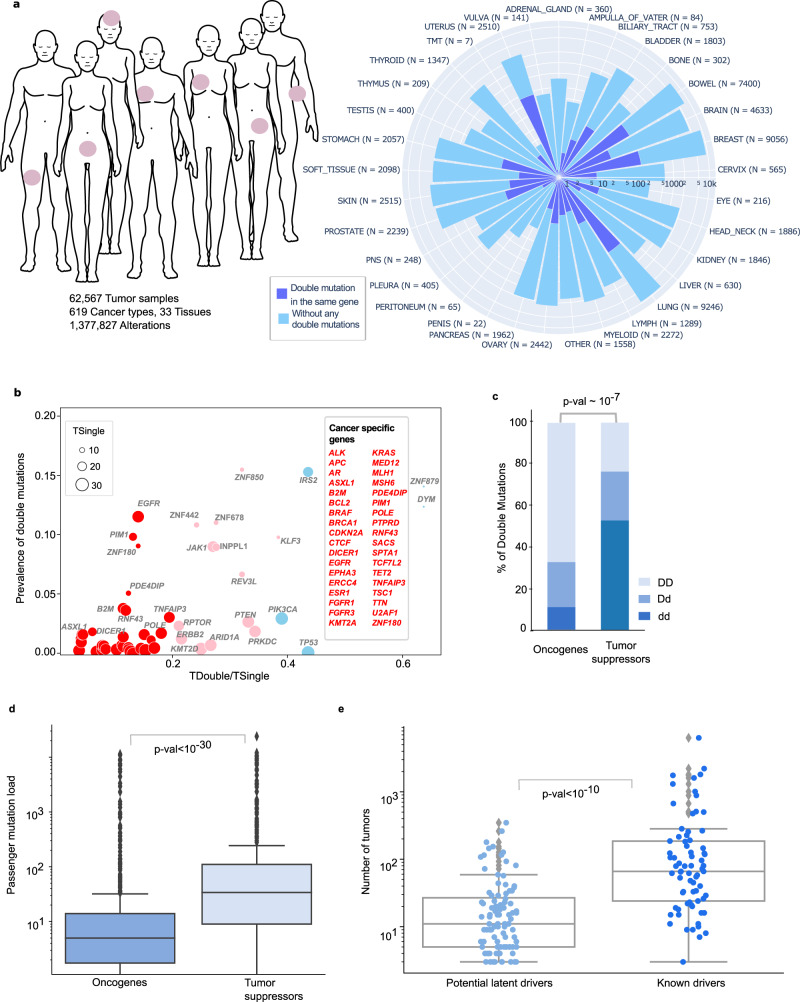
Overall statistics of the data, mutation load, and analysis of the significant double mutations. The data is filtered with variant allele frequency (VAF > 0.125). **a** Total number of tumors, alterations, cancer types in the union of TCGA and AACR GENIE studies (*n* = 62,567 tumor samples). Windrose plot shows the number of same gene double mutant (blue) tumors and without any significant double mutation (green) across 33 tissues on the log-scale axis. **b** Tissue specificity of same gene double mutations compared to their single mutant counterparts. Genes having cancer-specific double mutations are red and cancer-wide double mutations are in blue (25 TSGs with 72 double mutations, 13 OGs with 55 double mutations, and the remaining 15 are labeled as both or neither). **c** Composition of the double mutations based on known driver (D) and potential latent driver (d) labels in tumor suppressor genes and oncogenes where D is already known frequent driver mutations, d is relatively rare potential latent drivers (*p*-val < 10^−7^, two-sided Fisher’s Exact Test). **d** Box plot showing passenger mutation load in OGs and TSGs (*p*-val < 10^−30^, two-sided Mann–Whitney *U* test). **e** Tumor count distributions of known driver and potential latent driver mutations (*p*-val < 10^−10^, two-sided Mann–Whitney *U* test).

We exploited the mutation profiles from TCGA and GENIE pan-cancer cohorts to discover latent drivers (Fig. 1a). We included all non-synonymous mutations, including missense, nonsense, nonstop, and frameshift mutations. We excluded frameshifts (insertions or deletions) that alter more than one position in a protein. We also excluded variants where the wild type and/or mutant residues are not specified. Finally, we filtered out the mutations that have VAF (Variant Allele Frequency) less than or equal to 0.125 to assure that the mutations are present approximately in 25% of the sequenced tumor cells.

We identified potential double mutations from proteins having two or more mutations at different positions in the pan-cancer data. Pairwise combinations of mutations in the same gene are pooled and evaluated as potential double mutations. As a result, we obtained 2,230,203 potential double mutations to be tested among 62,567 tumors.

To assess the significance of all potential double mutations (2,230,203 doublets), we constructed a 2 × 2 contingency table for each pair of mutations in each gene (12,724 genes). We built the tables according to the number of samples where constituents of a double mutation are present together, only one of the constituents is present, and none of them are present. (see “Methods” section). Applying Fisher’s Exact Test followed by multiple testing correction (Benjamini–Hochberg, *q* < 0.1) resulted in 11,532 significant pairs. Then, we filtered out the doublets if both of the mutation constituents are nonsense (411 double mutations were filtered out of which 49 and 4 were in *APC* and *PTEN*, respectively; the rest scattered across 190 different proteins). A component in downstream of a nonsense mutation in a doublet is either a false positive (chance passenger with no functional consequence), or in *trans* (not a true double mutation affecting the same protein). Thus, we also filtered these significant double mutations out (1377 doublets where 80 and 15 are in *APC* and *PTEN*, respectively; the rest are on 552 different proteins). Then, we applied a stringent filtering to the rest to ensure that co-existing mutations are not erroneously identified. Given a mutation pair *(i, j)*, if mutation *i* is present in x_i_% cells and mutation *j* is in x_j_% cells and the total of x_i_ and x_j_ is greater than 100%, it is highly likely that double mutation components truly overlap in the cells. After filtering statistically significant doublets based on the proportion of nonsense mutations in double-mutant tumors and total VAF value of each double mutation in the corresponding tumor, 7252 significant doublets were identified, 155 of which were present in three or more tumors. We labeled the constituents true co-existing mutations and retained 155 double mutations for further analysis (“Methods” section, Fig. S1, and Supplementary Data 1).

We labeled double mutation components as known driver (D) if it is a validated oncogenic mutation in Cancer Genome Interpreter^9^; and otherwise potential latent driver (d). We classified a known driver mutation as a strong driver if it is present in more than 10% of the gene-mutant tumors; otherwise, it is a weak driver. Similarly, we dubbed a potential latent driver mutation as a strong latent driver if it is present in more than 1% of the gene-mutant tumors; otherwise, we classified it as a weak latent driver. Here, we propose that combinations of two strong latent driver mutations can act like a known driver, whereas weak latent drivers can only potentiate the effects of weak driver mutations. We classified mutation pairs as co-occurring based on the odds ratio (OR, log_2_(OR) > 0), and the rest as mutually exclusive. As a result, we identified 148 co-occurring and 7 mutually exclusive double mutations. The mutually exclusive doublets are composed of known driver mutations, i.e., the constituents are either weak- or strong- driver mutations.

We examined the *cis/trans* occurrences of the double mutation components. We used publicly available supplementary data from Saito et al., Gorelick et al., and Vasan et al. since raw data or allelic configuration information for the GENIE data, which constitutes around 90% of our dataset, is unavailable^29–31^. With the availability of raw data or allelic configuration information, it would be possible to enlarge the set of double mutations that are in *cis*. The analysis identified 36 tumor samples carrying double mutations matching our data. As a result, we could find *cis* and *trans* information in our double mutation dataset for only 19 doublets accumulated in six genes. For each of the 19 doublets, if the *cis* occurrence is higher among the double mutant group, we labeled it as *cis*, and *trans* otherwise. In total, 8 (5 *cis*, 3 *trans*) of these doublets are in the TSGs *PTEN* and *TP53*, the remaining 11 doublets are in OGs *EGFR*, *ERRB2*, *KRAS*, and *PIK3CA* where 10 are in *cis*; and one of them is inconclusive due to the equal number of *cis* and *trans* occurrence.

Recently, the frequency of driver genes was analyzed together with the maximum prevalence of their mutations, distinguishing cancer-specific drivers versus cancer-wide drivers^13^. We applied a similar analysis to our dataset composed of double mutations in the same gene where we obtained the ratio of the number of tissues carrying double mutations (*T*_double_) and single mutations (*T*_single_). We also calculated the prevalence of double mutations compared to single mutations. For example, *KRAS* double mutations are observed in tumors in four tissues (bowel, pancreas, skin, lung), but single mutations of *KRAS* can be seen in tumors from 30 different tissues. Thus, the tissue specificity, *T*_double_/*T*_single_, of *KRAS* is ~0.13. Prevalence of *KRAS* is the ratio of the number of double mutant tumors (*n* = 8) to the number of all *KRAS*-mutant tumors (*n*~8000), which is ~0.001. Values closer to the origin on the x-axis indicate tissue specificity since for each gene the number of double mutations carrying tissues is smaller compared to the number of single mutations carrying tissues. Larger numbers on the y-axis represent the multitude of patients with double mutations on the gene. Hence, double mutations of *KRAS* can be considered as tissue specific with a low prevalence. As a result, although some genes and their single mutant states have been previously cataloged cancer-wide, we found sets of double mutations that are cancer tissue-specific. Examples include double mutations in *BRCA1*, *EGFR*, and *KRAS* (Fig. 1b).

Double mutation components that are not known drivers can be considered as ‘potential latent driver’ mutations. In a doublet, the components can be known drivers or potential latent drivers, so each doublet is cataloged as DD, Dd, and dd. That is, DD is a known driver-known driver doublet, Dd is a known driver-potential latent driver and dd is a doublet consisting of two potential latent drivers. Among the 155 same gene double mutations, there are 54 DD, 29 Dd, 72 dd. The 155 same gene double mutations are composed of a pairwise combination of 213 mutations of which 73 are known drivers and 140 are latent drivers. Thus, our analysis can capture rare mutations that are potential latent driver candidates.

The 155 significant double mutations are composed of 213 mutations in 53 different genes. These 53 genes harbor 34,011 mutations that are observed in at least one tumor. Therefore, the fraction of double mutation components among all mutations (in 53 genes carrying at least one same gene double mutation) is ~0.6%. When we evaluate all mutations on 53 genes that are observed in at least three patients, the total number of such mutations is 6245 and the fraction is ~3.4% (Fig. S2).

When we solely examine the double mutations in genes classified as OG or TSG, the number of doublets of type DD, Dd, dd is 37, 12, 6, and 17, 17, 38 in the 13 OGs and 25 TSGs, respectively. We observe that OGs have significantly more DD mutations than TSGs (*p*-value < 10^−7^, two-sided Fisher’s Exact Test) and the fraction of double mutation components among all mutations in these 13 OGs (~1.2%) is almost two times higher than the fraction of double mutation components among all mutations in these 25 TSGs (~0.5%; Fig. 1c). This result implies that becoming more oncogenic requires mostly co-occurrence of two frequent mutations while suspending tumor suppressor activities may involve rare mutations coming together.

In the pan-cancer dataset, same gene double mutations accumulate in 53 genes, of which 25 are TSGs, 13 are OGs, 2 are both OG and TSG, and the rest (13 genes) are in other functional categories. There are 821 double mutant tumors carrying at least one double mutation in these 53 genes. In total, the number of tumors having at least one double mutation in an OG and TSG is 468 and 307, respectively. Patient tumors that have at least one double mutation in any TSG have a significantly higher passenger mutation load compared to patient tumors having at least one double mutation in an OG (*p*-value < 10^−30^, two-sided Mann–Whitney *U* test, Fig. 1d). Given that only ~2% of the 41,734 tumors (having at least one mutation in the 53 genes) carry a double mutation, double mutations are comprising a very small portion of gene-mutant tumors. Especially, TSGs require a very high mutation load for two coexisting mutations in a single gene. Based on the mutation load, and in line with our previous result, loss of function through double mutations in TSGs requires considerably higher mutational load compared to gain of function in OGs. We further compared the mutation load of TCGA and GENIE cohorts separately, taking into account the differences in coverage between the sequencing technologies (Figure S3). There are 63 and 69 tumors with at least one double mutation in an OG and TSG, respectively, in TCGA. Similarly, GENIE has 405 and 238 tumors with at least one double mutation in an OG and a TSG, respectively. Our finding that tumors with at least one double mutation in any TSG have a significantly higher passenger mutation burden is preserved in both the TCGA and GENIE datasets (two-sided Mann–Whitney *U* test, *p*-values 0.003 and 10^−30^, respectively). In addition, comparing passenger mutation loads among all tumors from TCGA (9588 tumors) and GENIE (52,979 tumors) revealed that TCGA tumors have a larger passenger mutation load (Fig. S3A, B).

Among the sample group harboring at least one double mutation in a TSG, both passenger and passenger+driver mutation loads are higher than in OGs (Figs. 1d and S4). Moreover, there are 43 known-driver and 22 latent driver mutations in OGs; and 30 known-driver and 74 latent driver mutations in TSGs when we compare the counts of the known-driver and potential latent driver mutations contributing to the formation of significant double mutations.

Double mutations in TSGs are more enriched in latent driver mutations compared to OGs. This abundance could be due to the higher passenger mutation load among tumors with double mutations in TSGs. Despite the small number of samples with *cis/trans* information, the double mutations in TSGs mainly occur in *trans*.

Despite several genes with a high rate of single mutations among double mutant genes in different tissues, there are few double-mutant genes that are tissue-specific. Additionally, in contrast to TSGs, the doublets in OGs are mainly comprised of known driver mutations. Double-mutant tumors with at least one TSG doublet have a higher passenger mutation load. These could be attributed to different mechanisms in elevating oncogenic signaling and lowering tumor-suppressive signaling through double mutations among OGs and TSGs, as well as their biological impact. Here, the task is to decide whether latent driver mutations in TSGs are functional or they are false positives due to the passenger mutation burden. There is a need for pre-clinical models such as patient-derived xenografts or cell lines containing double mutations in *cis* in TSGs. To inspect the role of such latent drivers, it would be enlightening to perform a comparative analysis of tumor growth or drug response in wild type, single and double mutant (in *cis*) pre-clinical models.

### Functional interpretation of double mutations by using the characteristics of their constituents and double mutant tumors

To interpret the functional consequences of double mutations, we elaborated on the frequencies of the mutations forming the significant pairs, the chemical properties of the wild-type and mutant residues, or the relationships of the double mutation components with mutational signatures. Known driver mutations have a higher frequency than potential latent driver mutations (Fig. 1e). The median values of tumor count for known driver and potential latent driver mutations are 70 and 9, respectively (*p*-val < 10^−10^, two-sided Mann–Whitney *U* test). Potential driver mutations are relatively rare, and their pathological impact can be dramatic when they couple with another mutation. Therefore, we cataloged all potential latent driver mutations that contribute to a significant doublet in the same gene as strong or weak latent drivers. The list of 140 latent drivers can be found in Supplementary Data 1.

Next, we followed a bottom-up approach to obtain the spatial, chemical, and pathway level organization of the double mutations. We used the pan-cancer mutation clusters deposited in 3DHotspot where each cluster represents the set of mutations that are spatially close to each other^34^. We found that components of the doublets in the same gene are usually spatially distant from each other. The simultaneous presence of two strong spatially close driver mutations is rare in a patient tumor; there are only 15 doublets belonging to the same cluster accumulated in *EGFR*, *KRAS*, *PIK3CA*, and *TP53*. However, some weak drivers are proximal to either a strong driver or another weak driver, as in the cases of mutations at positions R130/R173 in *PTEN*. Spatially close residues may form potent allosteric couples, which may enhance proliferation.

There are four rare (significant double mutations observed in less than three tumors) BRAF doublets (Supplementary Data 1). Here, the mechanisms of BRAF mutations were classified into those suggested to be activated as monomers (Class 1), acting as constitutive active dimers (Class 2), and those having impaired/dead kinase activity (Class 3)^35^. There are two doublets having a mutation from Class 2 (K601, L597). These rare double mutations are still kept when we apply a more stringent threshold for total VAF value (up to 40%).

During the formation of double mutations, we had assumed all mutations at a specific position in a protein as the same mutation. We traced back to the mutation positions and obtained wild type and mutated amino acid types to obtain the chemical class changes. A comparison of the fraction of chemical classes of the wild type and mutant residues revealed that Charged>Polar and Charged>Charged switches are more dominant among TSGs and OGs, respectively (*p*-values ~0.009, 0.04 respectively, two-sided Fisher’s Exact Test, *p* = 0.05; Supplementary Note and Fig. S5A). Similarly, for the double mutation components that are known Driver [D] or potential latent driver [d], we compared the chemical class alterations of the mutations. Hydrophobic>Hydrophobic changes are more common among tumors carrying potential latent drivers. Charged>Polar and Charged>Charged changes are prominent among tumors carrying known drivers (Fig. S5B).

In total, the number of tumors having at least one double mutation in an OG and TSG are 468 and 307, respectively. The distribution of variant classifications among the tumors carrying at least one double mutation in an OG is as follows: missense+missense (~99%), missense+frameshift (<1%), missense + nonsense (<1%; Fig. S6A). Doublets with both mutation components being missense mutations predominate among these tumors. On the other hand, we see a more diffuse result when we analyze the tumors harboring at least one double mutation in a TSG (Fig. S6B). These tumors have variant classifications as missense + missense (50%), frameshift+frameshift (~30%), missense+nonsense (~10%), missense + frameshift (~3%), and frameshift + nonsense (~0.48%). The sample-specific details related to variant classifications of double mutations in OGs and TSGs are provided in Supplementary Data 1.

During post-processing, we identified 3519 tumors as hyper-mutated out of 62,567 samples with at least one point-mutation with a Q3 + 8 x IQR threshold (see “Methods” section). First, we used Fisher’s Exact Test (*p* < 0.05) to test the robustness of the 155 double mutations against hyper-mutated samples. Hyper-mutated samples carry 19 doublets in several genes including *APC*, *KMT2D*, *ZNF442*, and *ZNF678*; therefore, we excluded these doublets from the subsequent analyses. Among the remaining 136 doublets, one is not significant according to Fisher’s exact test (*p* < 0.05) evaluated in the non-hyper-mutated tumor group (Supplementary Data 2).

Then, we performed a permutation test (*p* < 0.01) using the non-synonymous mutation burden of the double-mutant and single-mutant tumors (see “Methods” section). For each double mutation, we tested the null hypothesis that the double mutant tumors (labeled “Double”) have a mean mutation burden less than or equal to the mean mutation burden of the remaining gene-mutant tumors (labeled “Single”). We can reject the null hypothesis for 7 doublets since the p-values obtained with the permutation test are <0.01. For the remaining 129 significant doublets the evidence is not sufficient to conclude that the double mutant tumor samples have a lower or equal mean observed mutation load on the basis of failure to reject this as a null hypothesis (Supplementary Data 2)

We next conducted single base substitutions (SBS) signature analysis of double mutations to explore if components of doublets have common or different signatures (a.k.a. contexts). There are 96 single base substitutions (SBS) of the trinucleotide context where the mutated base is in the middle in square brackets expanded with 5’ and 3’ bases^36^ (e.g. T[G > A]A). We only considered missense mutations in SBS analysis. As a result, we analyzed 711 records (tumor-specific information for each doublet) from 115 doublets in 649 tumors. Within this set, the majority of the double mutations are of different contexts (630 records), and all of these records match with one of the 96 contexts (see “Methods” section). There are 81 records (composed of 17 doublets in 77 tumors) where the double mutations are of the same context. The contexts T[G > A]A, C[G > A]A, C[A > G]T, and A[G > T]A are dominant and are present in 40, 13, 5, and 5 records, respectively. Doublets from the same context are mostly located in *PIK3CA* (Supplementary Note and Supplementary Data 3).

Taken together, double mutations are exceedingly rare phenomena and do not positively correlate with the tumors’ mutation burden. The components of the doublets that have been classified as latent driver mutations also occur far less frequently than known driver mutations. The chemical classes of the wild-type and mutant amino acids as well as the variant classes of the doublet constituents are different among the double mutations in OGs and TSGs.

### Doublets in the same gene are rare but are a signature for some cancer types

**Fig. 2:**
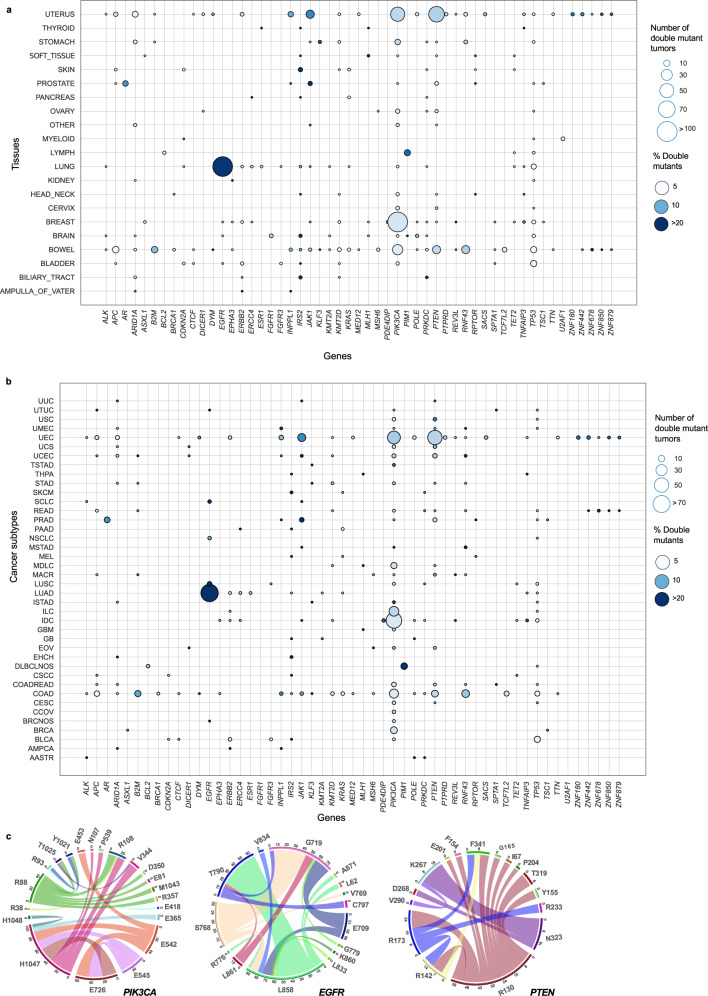
Same gene double mutations are specific to some tissues or cancer subtypes. Bubble plots show number (node size) and frequency (node color) of double-mutant tumors among gene-mutant tumors across different tissues and cancer subtypes (Oncotree). For the 53 genes with significant same gene double mutations, node size represents the number of patients carrying at least one doublet in a gene in a tissue or cancer type. **a** Presence of same gene double mutations across different cancer tissues where at least three tumors carry at least one same gene double mutation in one of the 53 genes. **b** Presence of same gene double mutations across different cancer subtypes. The cancer subtypes where at least five tumors carry at least one double mutation are listed on the *y*-axis. **c** Representation of mutations in genes to compose a doublet as a circular diagram. The strips from one residue to another represent significant double mutations with size of strips indicating frequency of each double mutation.

After identifying the doublets that are significant at the pan-cancer level, we also assembled tissue-specific sets of double mutations since tissues differ in sample size and are enriched in different genes and mutations. Identification of tissue-specific double mutations are particularly essential because they may point to the tissue of origin of the preclinical models to evaluate drug responses and tumor growth patterns. As shown in Fig. 2a, co-occurring double mutations in the same gene are relatively rare, with varied frequencies across tissues. In some tissues, doublets are present in the same gene in up to 10% of the patient tumors (e.g., bowel and breast tissues). However, same gene doublets are either extremely rare or not present in other tissues, such as the pancreas, ovary, liver, kidney, and biliary tract.

Since double mutations are significantly less common than single mutations (*t*-test, *p*-value~0.006), tissue-specific double mutations can have important roles to predict sensitivity/resistance to specific inhibitors. Here, we aimed to determine the fraction of tumors with at least one double mutation in the corresponding gene among all gene mutants in each tissue or cancer type. Fig. 2a illustrates the tissue-specific prevalence of double mutations in the same gene. *TP53* and its double mutations are cancer wide. *PIK3CA* double mutations are quite common in breast and uterus tumors. Among lung tumors, *EGFR*, and among bowel tumors, *PIK3CA* double mutations are ahead by far. Bowel, breast, and lung tissues are enriched with double mutations in specific genes whereas brain tissue has significant but rare double mutations in multiple genes such as *FGFR1*, *IRS2*, *POLE*, and *TP53*. LUAD (Lung Adenocarcinoma) is enriched with *EGFR* double mutations. COAD (Colon Adenocarcinoma) is enriched with *B2M*, *PTEN*, and *RNF43* double mutations. We note that *PIK3CA* double mutations are relatively more dominant in BRCA, IDC (Breast Invasive Ductal Carcinoma), ILC (Breast Invasive Lobular Carcinoma), COAD, and UEC (Uterine Endometrioid Carcinoma) subtypes (Fig. 2b).

The most frequent mutation, G12D on *KRAS*, is rarely coupled with another mutation in *KRAS* (Supplementary Data 1). The mutational mosaic of *KRAS* is distinguishable among different cancer types. G12D is predominantly present in pancreatic, lung, and colorectal cancers. *KRAS* mutations are context specific, and a mutation may act differently in different cancer types.

*PIK3CA* has three driver mutations- H1047, E45, and E542- mostly accompanied by a group of rare mutations that are potential latent driver mutations. Along the same lines, the driver mutations L858, T790, G719 on EGFR; R130 and R173 in *PTEN* have rare potential latent driver mutation companions (Fig. 2c).

Thus, even though rare, doublets on the same gene can be a signature for some cancer types, e.g., bowel, breast, and lung cancers.

### Linking double mutations to clinical data using cancer cell lines and xenografts

We next explored the potential clinical association of the significant same gene double mutations. Since the patient-specific clinical and treatment data are sparse, we computationally screened differences in cell lines and patient-derived xenografts (PDXs) from the experimental datasets. We used cancer cell lines from the DepMap project and PDX samples provided in Gao et al.^37^. In both datasets, mutation profiles and response to a panel of hundreds of drugs are available. Double mutations are rare in the patient tumor samples. We notice the same pattern: Despite scanning hundreds of cancer cell lines, double mutations in the same gene are rare. Among 155 same gene double mutations only 23 double mutations are present in at least one cell line in Cell Model Passports^38^. The intersection between the significant double mutations and their presence in the cell lines led us to pursue a detailed analysis on the genes *PIK3CA* and *EGFR*.

**Fig. 3:**
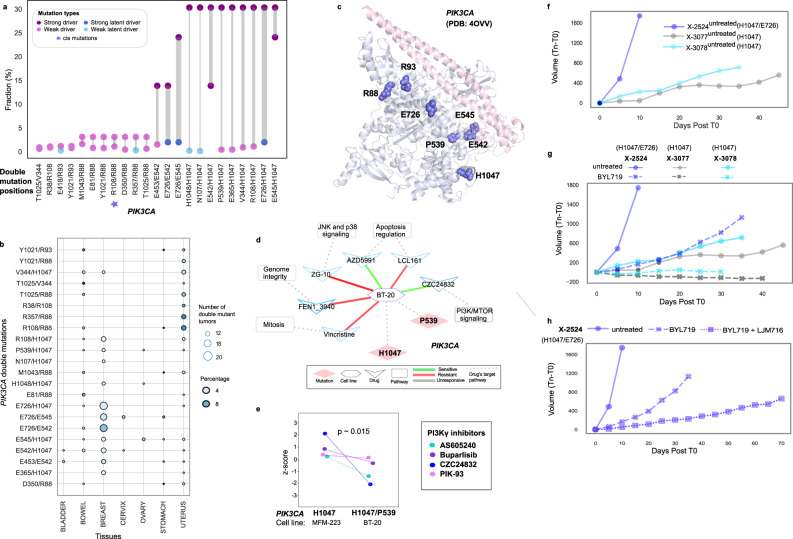
A detailed analysis of *PIK3CA* double mutation profile, 3D structure, and clinical implications. **a** Paired dot plot of the 23 double mutations in *PIK3CA*, and the number of tumors carrying them. Colors indicate type of a mutation, strong driver (purple), weak driver (orchid), strong latent driver (blue), and weak latent driver (sky blue). Line sizes connecting the dots are proportional to the number of tumors with double mutations. **b** Presence of *PIK3CA* same gene double mutations across different cancer tissues. Dots are scaled based on the number of tumors having double mutations, and color corresponds to the percentage of double mutant tumors among single mutants. **c** 3D structure of *PIK3CA* (PDB: 4OVV) with H1047, E726, E542, E545, R88, R93, and P539 mutations. **d** Response of *PIK3CA* breast cancer cell line BT-20 with double mutations in *cis* to drugs in network representation. Dashed lines connecting cell line nodes (hexagon) to mutation nodes (diamond) indicates that the cell line harbor the corresponding mutations. Green and red lines connecting cell line nodes (hexagon) to drug nodes (V-shaped) represent sensitivity and resistance of the cell line to the corresponding drugs, respectively. Dashed lines between the drug nodes (V-shaped) and pathway nodes (rectangle) indicate that the drugs have target(s) in the corresponding pathways. **e** *PIK3CA* mutation doublets in breast cancer and the associated violin plot illustrating response to PI3Kɣ inhibitors. **f** Tumor volume changes of single and double *PIK3CA* mutant xenografts without any treatment. **g** Tumor volume comparison of the single and double mutant xenografts without any treatment and with BYL719 (Alpelisib) treatment. **h** Comparing tumor volume changes of the double *PIK3CA* mutant xenografts without any treatment and with BYL719 and BYL719 + LJM716 treatment.

*PIK3CA* has both strong drivers (e.g., H1047R, E545K), and weak drivers (e.g., R88Q, E453K, M1043I) which are components of 23 significant double mutations in the patient cohort. Despite *PTEN*, *TP53*, *EGFR* and the rest (53 genes in total) have a higher single mutation load compared to *PIK3CA*, their double mutation load is by far less (Fig. S7). Full activation of oncogenic *PIK3CA* is through at least two drivers acting in different, albeit complementary mechanisms, or enhancing each other. One well example of how the co-occurrence of in *cis* mutations might promote cancer is PI3Kα^29,31^. Moreover, crystal structures and experimental research have shown the activation mechanism at the atomic scale, and the role of frequent or rare driver mutations on this mechanism is widely discussed^39–43^. E542K, E545K, and H1047R are hotspot helical and kinase domain mutations that can activate PI3Kα, but they can also have additive effects when combined with the moderate mutations E453K/Q, E726K, and M1043V/I^25,43^. Sporadic and weak activating mutations in PI3Kα are also present. The weak mutations cause conformational changes that lead to PI3Kα activation. These weak mutations include E726K and M1043V/I in the kinase domain, N345K, C420R, and E453K/Q in the C2 domain, and R38H/C, R88Q, R93Q, R108H, and G118D in the ABD domain^43^. Thus, the pathological impact of a single driver may be insufficient^44^. One well-known example is H1047 and E545 double mutation enhancing proliferation. However, E545 and E542 double mutations do not make *PIK3CA* reach the fully activated level. A combination of two strong latent driver mutations – but likely not two weak mutations – may act like a driver mutation. The frequency of double mutation components in *PIK3CA* is shown in Fig. 3a where many doublets are composed of one frequent and one rare mutation^39,44^.

Our frequency-based analysis revealed that E726 is a potential strong latent driver while N107, R357, E418, and H1048 might be weak latent drivers coupled with a weak or strong driver mutation. *PIK3CA* double mutations are also tissue- and context-specific as shown in Fig. 3b. Most are in breast tissue. An exception involves doublets consisting of R88Q which are depleted in breast but frequent in uterus tumors. Their structural location is shown in Fig. 3c. Kinase mutations work by destabilizing the inactive or stabilizing the active state. These are better captured by their detailed conformational alterations. A detailed analysis of the folding free energy (ΔΔG) upon double or single mutation with DynaMut^45^ shows the increased impact of several double mutations in the protein stability (Supplementary Note and Fig. S8).

The impact of co-occurring mutations in the same gene is mostly additive but can be also cooperative. When the double mutant phenotype incorporates traits from the single mutants, it can be regarded as additive. Additivity is considered to be a sign that there is no functional link between the driver mutations under evaluation. When the combined effect of two mutations on the phenotypes is greater than the total of each mutation’s individual effects, they are referred to as cooperative (also known as synergistic, positive epistasis, or more-than-additive). But rather than just adding up the impacts of two mutations, it is possible to obtain a lesser effect (suppressed, negative epistasis)^32,46,47^. There are seven allosteric mutations at positions 83, 88, 365, 539, 542, 603, 629 in *PIK3CA* in BRCA as cataloged in Allosteric DB^48^. In total, 13 out of 23 *PIK3CA* double mutations are harbored by at least one breast tumor and there are 215 double mutant tumors. The doublet P539/H1047 in *PIK3CA* is composed of one strong driver (at position 1047) and one weak driver mutation (at position 539) which is allosteric. Their effects are additive.

We found a breast cancer cell lines with *cis* mutations^29^ in *PIK3CA* belonging to the BRCA subtype: BT-20 has P539/H1047 double mutation. H1047R is a frequent driver. However, P539 is a rare mutation in the pan-cancer data, making it a potential weak driver. To illustrate the difference between the double mutations and single mutations in terms of drug response, a network of cell lines to drugs and target pathways is constructed (Fig. 3d) where drugs are linked to each cell line which has altered response compared to their single mutant counterparts. Indeed, there is a difference in the response to PIK3α inhibitors in double-mutant cell line BT-20 compared to single mutant cell line counterparts (*p*-value = 0.015). Additionally, a double mutant BT-20 cell line is remarkably sensitive to the PIK3*γ* inhibitor CZC24832 while the single mutant MFM-223 (H1047) cell line does not show a remarkable response (Fig. 3e). Despite factors contributing to the drug sensitivity including other single point mutations and gene copy numbers, double mutations in *PIK3CA* may be still an important contributor as evidenced by increasing its oncogenic activity described in the literature. Therefore, we further explored PDX data to compare double mutant and single mutant *PIK3CA* tumors in terms of the tumor volume changes and drug responses. We found two PDXs having double PIK3CA mutations (E726/H1047, R88/T1025). In PDX X-2524 with doublet H1047R/E726K, a strong known driver/strong latent driver combination, the volume change of the tumor between days 0 and 10 is more than 1700 mm^3^, while single mutant tumors X-3077 and X-3078 (with mutation H1047R) have volume change of ~200 mm^3^ in the first 10 days reaching ~400 mm^3^ at around 35 days (Fig. 3f). The double mutant PDX tumor has a dramatically higher growth rate. We compared the growth pattern of double mutant PDX with only single H1047 mutant PDX since there was not any single E726 mutant PDX within the data set.

Tumor growth rate data of these three PDX tumors are also available for drug treatment. BYL-719 (Alpelisib) treatment, a selective PI3Kα inhibitor, diminishes tumor volume by 88% (~1600 mm^3^) in the first 10 days in the double mutant PDX (X-2524) which is dramatically higher than the single mutant PDXs (X-3077 and X-3078) implying increased drug sensitivity (Fig. 3g). Drug combination of BYL-719 with LJM716, an anti-HER3 monoclonal antibody, is even more effective in reducing tumor volume than BYL-719 treatment alone because of the HER3 alteration in this PDX (Fig. 3h). In cis E726K/H1047R doublet may be a potential strong driver of faster tumor growth rate and better response to PI3K inhibitor Alpelisib; however, no causal conclusions can be drawn without functional data for these cell lines and PDXs. Several factors may lead to this difference in tumor growth rate and response to PI3K inhibitor. Despite other alterations, these PDX models have only one known driver mutation (cataloged in Cancer Genome Interpreter) at position 1047 in PIK3CA and common in all three xenografts (X-2524: *PIK3CA* 1047/726 double mutant, X-3077 and X-3078: *PIK3CA* 1047 single mutant). Another factor is the copy number of the genes in PI3K/Akt/mTOR pathway which could affect *PIK3CA* activity, and drug response. The copy number values (the median values for individual exons called by ExomeCNV^37^) of *PIK3R1* and *AKT3* in the double mutant xenograft are two-fold higher than the single mutant samples (double mutant: 2.41, single mutants:1.34, 1.41). *PIK3R1* functions as a negative regulator of *PIK3CA*. Increased level of *PIK3R1* may negatively regulate the excessive activity of double mutant *PIK3CA*.

On the other hand, not all tumors having double mutation in *PIK3CA* show a similar pattern. For example, growth rate of the tumor (X-3093) with R88/T1025 is slower than of the tumor having a single mutation (at position R88), because both mutations are potential weak drivers and mutations in *PTEN* (E7 and R130*) in addition to other alterations. A tumor with only the R88 mutation is more responsive to PI3K inhibitors compared to that with R88/T1025 (Fig. S9A–H). *PTEN* is a tumor suppressor and *PIK3CA* is an oncogene. Active PI3K phosphorylates signaling lipid PIP_2_ to PIP_3_. This activates a cascade of protein kinases leading to the cell cycle. *PTEN* suppresses cancer by dephosphorylating PIP_3_ back to PIP_2_. Loss of function at *PTEN* and gain of function at *PIK3CA* ascends PIP_3_ levels in the cells^49^. *PTEN* is a negative regulator of the PI3K/Akt/mTOR signaling pathway. Overactivation of *PIK3CA* and loss of activity of *PTEN* due to the double mutations can lead to hyperactivation of PI3K/Akt/mTOR signaling which may result in oncogene induced senescence (OIS), potentially explaining the blockage of tumor growth in the double mutant X-3093 xenograft.

**Fig. 4:**
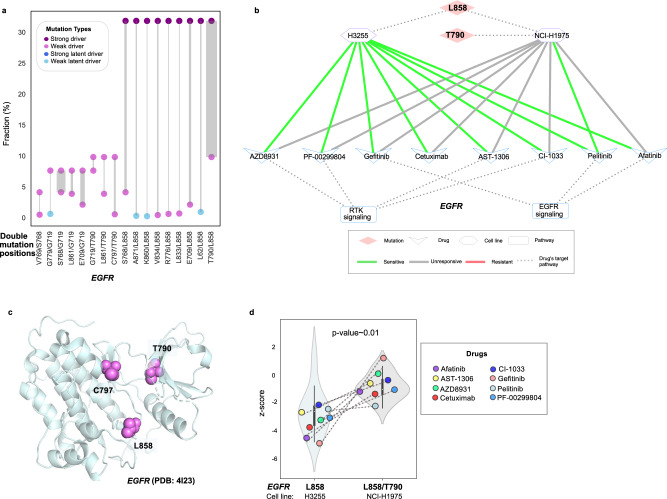
Structural and clinical aspects of EGFR double mutations. **a** Paired dot plot of *EGFR* double mutations. Each paired dot represents one double mutation. Dots are colored according to their type, driver (purple), weak driver (orchid), strong latent driver (blue), and weak latent driver (sky blue). Line size connecting the dots is proportional to the number of tumors with double mutations. **b** *EGFR* mutation doublets in lung cancer cell lines and their response to drugs in network representation. Dashed lines connecting cell line nodes (hexagon) to mutation nodes (diamond) indicate cell lines that harbor corresponding mutations. Green and red lines connecting cell line nodes (hexagon) to drug nodes (V-shaped) represent sensitivity and resistance of the cell lines to the corresponding drugs, respectively. Dashed lines between the drug nodes (V-shaped) and pathway nodes (rectangle) indicate that, the drugs have target(s) in the corresponding pathways. **c** Representation of double mutations in *EGFR* structure. **d** EGFR mutation doublets in lung cancer together with the violin plot that shows the response to RTK inhibitor in double mutant and single mutant cell lines. More negative *z*-score means more sensitivity and more positive *z*-score means more resistance to the drug molecule.

Another oncogene with latent driver mutations is *EGFR*; the mutations L62, G779, K860, and A871 are weak latent accompanied by weak/strong driver mutations (Fig. 4a). There are 17 double mutations in *EGFR*; these doublets are mostly composed of weak drivers (7 doublets) and weak+strong driver combinations (6 doublets).

A combination of a weak driver and a strong driver mutation T790/L858 double mutation in *EGFR* is present in one cell line (NCI-H1975) of lung cancer. H3255 cell line has only one mutation at position L858 in *EGFR* (Fig. 4b). Both mutations are in the kinase domain to which the RTK inhibitors bind (PDB: 4I23, Fig. 4c). However, response to the inhibitors is considerably different in the cell line with double mutant *EGFR*. It is more resistant compared to the single mutant cell line (*p*-value ~ 0.01, two-sided Mann–Whitney *U* test, Fig. 4d).

L858R in *EGFR* is sensitive to *EGFR*-targeted tyrosine-kinase inhibitors (TKIs). After treatment with TKIs, T790M, has been observed. It decreases TKIs’ binding^32,50^. L858R lies in the A-loop of the drug binding pocket and destabilizes the inactive conformation. The “gatekeeper” residue T790M is in the hinge region of the binding pocket. L858/T790 increases the protein stability and changes the conformation of the binding pocket which generates resistance to the *EGFR* inhibitors^51,52^. Another double mutation is T790/C797. The sensitivity of the T790M mutant lung cancer tumors to the third generation TKIs vanishes with the emergence of C797S^32^.

Collectively, pre-clinical models -PDXs and cell lines- bearing double mutations show different growth and drug response patterns. The *PIK3CA* double-mutant PDX grows faster, and its growth trend differs from the single-mutant PDXs. Better response to the PI3K inhibitors both in double mutant cell lines and PDX give clues to their clinical behavior, despite the necessity of functional data. On the contrary, *EGFR* double mutation may lead to increased resistance by altering the inhibitor binding pocket. Overall, the double mutations and single mutation counterparts are not the only genetic difference between pairs of single mutant and double mutant cell lines or PDXs. However, the prominent difference between double and single mutants in terms of drug response and tumor growth make them good candidates for further exploration of their clinical association.

## Discussion

In this study, we scan the cancer genome landscapes aiming to identify latent drivers. In our definition, mutations which are statistically frequent and thus labeled as oncogenic hotspots in the literature are strong drivers. Oncogenic mutations in the long tails of the distributions are statistically rare. They can be strong or weak drivers. Mutations that are rare^25^ and not yet labeled as oncogenic can be latent drivers. They may or may not be allosteric^53^. Rare drivers can be as potent as frequent drivers. Their low statistical frequencies may simply be an outcome of the computational strategy that has been employed in the calculation^54,55^. They may be tissue, or cell specific, harbored in specific cancers. Apart from repressors, under physiological conditions, the wild-type inactive state is more highly populated than the active state. Driver mutations, whether frequent or rare, destabilize the inactive state and/or stabilize the active state making the active state more populated than the inactive state. Two or multiple driver mutations can destabilize the inactive state to a greater extent than single driver mutation as compared to the active state, shifting the population toward the active state. Especially, the conformational change that they promote may also involve steric hindrance at the drug binding site. However, an allosteric mutation away from the binding site may restore drug efficacy against highly resistant mutants, as observed in *BCR-ABL1*^56^. A latent driver also either destabilizes the inactive state and/or stabilizes the active state, but the relative difference between the states can be smaller. Consequently, on their own their contribution to protein activation is relatively small, hindering their identification. However, the additive contributions of strong drivers or of latent drivers (strong or weak) can increase the population of the active conformations leading to the ensemble being fully activated. Given that the mechanism described here depends on the positions of the constituents in the 3D protein structure and their distance from one another in addition to other factors, it is entirely plausible that it cannot apply to all doublets.

We designed this study to discover latent driver mutations based on the premise that *in cis*, latent, and weak mutations can cooperate to enhance the oncogenic signal. We identified 155 significant, same gene double mutations which are composed of mostly one rare and one frequent. Frequent mutations have been cataloged as strong drivers^4,5,25^. Rare drivers can also be strong drivers. We newly cataloged 140 latent drivers. Even though they may be cancer-wide, coupling with another mutation increases their cancer-type specificity. The load of double mutations in tumor suppressors is significantly higher than in oncogenes, indicating their relative robustness to functional loss.

With the sparsity of patient treatment datasets, cell lines or patient-derived tumor xenografts are a useful clinical interpretation resource. We found evident differences in the response to PI3K inhibitors in tumor models that differ in the presence or absence of double mutations in *PIK3CA*, which is in line with recent experimental work^29^. Tumor growth is extremely fast in double mutant *PIK3CA* compared to the single mutant. Recent mechanistic studies suggest that the increased protein activity or acquired drug resistance is due to the mutation combinations. Zhang et al.^44^ suggested that combinations of strong and weak drivers can enhance PI3K activity and explain the phenotypic differences in *PIK3CA* double mutant tumors^43^ that we observed prominently in breast and uterus tumors. Here we further extended the analysis to combinations of less frequent mutations not cataloged as drivers, which we view as potential latent drivers. Among them, doublets with mutation at position R88 are depleted in breast but not in uterus cancers, suggesting that potential latent driver mutations pairing with R88 are important signatures of uterus tumors.

Not limited to *PIK3CA*, numerous other significant double mutations with possible prognostic or therapeutic impact have also been identified (i.e., *EGFR* in the lung in line with previous studies^30^). To fully understand mutational frequencies requires detailed functional data related to specific mutations, their combinations, and the proteins that harbor them. We, and others, have been aiming to reveal the mechanisms of oncogenic mutations in key protein nodes in the network. The paramount principle that guides us is that the mechanisms of the mutations mimicing the physiological activation^57^. However, whereas physiological activation is regulated, taking place following some signaling event, e.g., hormone binding to the extracellular domain of a receptor tyrosine kinase in the case of PI3K, with the signaling propagating downstream through a series of cascading events, oncogenic activation is dysregulated. We thus suggest that the single mutations which are components of doublets can act in one of two ways: their effects can be complementary in relieving the autoinhibition^4,58,59^, or can enhance the same effect, for example involving not one positive charge but two for membrane binding. Consider for example PI3K, whose physiological activation involves binding of the phosphorylated C-terminal motif of insulin receptor to the nSH2, resulting in breaking of the interaction of the nSH2 with the helical domain and relieving the autoinhibition, and binding of active Ras, which assists in binding and properly positioning the PI3K on the membrane. E542K and E545K hotspots mimic the action of the first, and H1047R the second. With all being strong hotspots, their co-occurrence can trigger oncogene induced senescence (OIS). However, a combination with more moderate mutations can powerfully activate this lipid kinase. Relieving the autoinhibition is a common physiological activation mechanism that oncogenic mutations adopt^58^. Not all mutations form pairs. One example is *BRAF* V600E. This has been attributed to its being a strong hotspot. Mutant *BRAF* V600E has been postulated to be activated as a monomer independent of Ras activation^60^ and shown to be able to phosphorylate MEK^61–63^. However, as we noted above, recent data suggest that even though the mutant is activated as a monomer, a dimeric BRAF is still required to phosphorylate MEK in cells^62,64–66^. Mechanistic arguments clarify that despite being an activating mutation for cell growth, BRAF V600E still requires a collaboration with a Raf partner to have MEK appropriately positioned and retained in the assembly, just as in the case of physiological *BRAF*^64^, an observation which is of vital importance in drug discovery aiming at targeting dimerization. This example serves to illustrate the importance of knowledge of the functional activation mechanism which statistics alone is unable to provide^67^. Combined, they may better forecast treatment outcomes. The sensitivity or responsivity of drug action to a targeted cancer therapy depends on how much the tumor relies on the particular oncogene and the cellular pathway with which it is associated. In *PIK3CA*, a combination of a driver mutation with a weak driver, or strong latent driver, particularly under different mechanisms of actions, have a good, albeit temporary, therapeutic response.

A major observation from our comprehensive analyses is that doublet mutations are infrequent events. We attributed the relative rarity of strong doublet hot spots to OIS. Another highly plausible explanation is that our doublets count identical mutations. However, the doublets can consist of mutations of similar chemical character. Mutations can emerge during cancer development to form doublets; however, commonly they pre-exist in the background mutational load. In contrast to rare strong hot spots, latent drivers may require additional collaborative mutations. Since their clinical or biological outcome is too weak to be observed, or the cells that harbor them may constitute a rare population, to date they were not considered in the patient cancer-specific protein sequence analysis. Alternatively, they may be silent, but a cryptic splice site which is executed may promote their expression^68^.

Our results, supported by drug response data of cell lines and patient-derived xenografts provide a strong background for therapeutic potentials of double mutations. Our results may form a basis for further experimental evaluation of molecular alterations to be exploited for therapeutics across different cancer types and in clinical identification. Mechanistically, the actions of *same gene* double mutations are more straightforward to interpret as compared to double mutations in different proteins in independent pathways. How double mutations in independent pathways work is still highly challenging to understand.

## Methods

### Data collection and processing

All available somatic missense mutation profiles are downloaded from The Cancer Genome Atlas (TCGA) and the AACR launched Project GENIE (Genomics Evidence Neoplasia Information Exchange)^69–71^. The TCGA mutation annotation file contains more than 10,000 tumors across 33 different cancer types. We used the merged MC3 file to get TCGA pan-cancer data. The somatic variants without sufficient normal depth coverage and variants found in the panel of normal samples were evaluated as possible germline variants and were removed from the file before merging.

The GENIE mutation file (Release 6.2-public) contains 65,401 patients and 68,897 tumor samples across 648 cancer subtypes under the Oncotree classification. Within the GENIE cohort 2930 patients match with multiple tumor barcodes. For those cases, only one primary tumor barcode is kept when available; if not, only one metastatic or unspecified tumor barcode is kept for further analysis without any other constraint. Among these patients, 2019 has sequenced primary tumors, 757 have sequenced metastatic tumors and the remaining 154 have tumors of the type not specified.

Next, we selected non-synonymous mutations including missense, nonsense, nonstop and frameshift mutations (altering only one position on a protein). We also excluded the mutations where the wild type and/or mutant residue name is not specified. As a result of this filtering process, 9703 and 57,921 tumors remained with a total of 1,631,755 point mutations in the TCGA and GENIE cohorts, respectively.

We did a pre-filtering on the VAF (Variant Allele Frequency) value to control the heterogeneity of the samples to some extent given that variants were collected by bulk sequencing in both datasets. We calculated VAF by using the ratio of the values in the t_alt_count and t_depth columns of the MAF (Mutation Annotation File) file of the pan-cancer data sets. Then, we continued our analysis with the mutations that have VAF value more than 0.125, ensuring that the remaining mutations are present roughly in 25% of the sequenced cells. We continued the analyses with 62,567 samples (9588 and 52,979 samples from the TCGA and GENIE cohorts, respectively) from 619 cancer subtypes and 33 tissues (including OTHER category).

### Statistics and reproducibility

We set pre-filtering criteria to find significant double mutations. This pre-filtering consists of total number of occurrences and VAF values of each individual mutation. We construct potential double mutations to be tested after prefiltering. Therefore, it is independent of the test statistic under the null hypothesis^70,71^. If an individual mutation is present in less than three tumors in the cohort and have a VAF less than 0.125, we filtered them out. We continued our calculations with the remaining 65,872 mutations on 12,724 genes, and for each gene and the mutations they are harboring in the final set we formed binary combinations. As a result, we obtained 2,230,203 potential double mutations to be tested in 62,567 tumor samples that have at least one point mutation with VAF > 0.125 and assessed their statistical significance (Fisher’s Exact Test). For each potential double mutation, we created a contingency table [[a,b],[c,d]] where a is the number of tumors having both alterations, b is the number of tumors having only the first alteration, c is the number of tumors having only the second alteration and d is the number of all tumors not having these two alterations together (*d* = 62,567−(a + b + c)).

Then, we applied multiple testing corrections by using Benjamini–Hochberg method and continued subsequent analyses with doublets having *q* < 0.1 and kept 11,532 significant doublets out of 2,230,203 potential doublets after multiple testing corrections (Supplementary Data 4)

We applied more filtering for the significant double mutations based on the nonsense mutation composition among double mutants and the VAF values of the constituents. Throughout our analyses, we assumed point mutations occur at the same position as same regardless of the mutant residue. We evaluated the VAF values of the components and the presence of a nonsense mutation in the upstream in a tumor-specific way for the significant double mutations and double mutant tumors. In the components of a doublet, despite having a mutation at the same position, the mutated amino acid may result in a missense, nonsense or frameshift mutation. Therefore, a double mutation can be one of the combinations of the following variant classes: missense+missense, missense + frameshift, missense+nonsense, frameshift+frameshift, nonsense+nonsense. Among the tumor barcodes having a double mutation, if at least half of the barcodes carries a nonsense mutation as a component of a doublet we filtered them from our dataset.

To inspect whether double mutation constituents are in the same set of sequenced cells in a tumor, we first calculated the total VAF value of double mutation components. If the total VAF value is >0.5, mutation components encompass >100% of the sequenced cells, which is impossible unless there is an overlap. Therefore, we labeled the mutation constituents as highly likely overlapping for such records. We retained the double mutations where the constituents overlap for at least 20% of the records for further inspection and kept 7252 significant double mutations where 155 of them are present in at least three tumors.

We used the Catalog of Validated Oncogenic Mutations from the Cancer Genome Interpreter^9^ to label double mutation components: if a mutation is among the 5601 driver mutations, we labeled it as known driver (D), otherwise potential latent driver (d). For each gene harboring at least one double mutation, we collected all the tumors with mutations present on at least 3 tumors as gene-mutant tumors. Then, we calculated the fraction (%) of tumors with double mutation components among the gene-mutant tumors. We classified a known driver mutation as a strong driver if it is present in more than 10% of the gene-mutant tumors; otherwise, it is a weak driver. Similarly, we dubbed a potential latent driver mutation as a strong latent driver if it is present in more than 1% of the gene-mutant tumors; otherwise, we classified it as a weak latent driver. Here, we considered mutations in each gene present in at least three tumors when generating gene-mutant tumor groups.

Additionally, double mutations are annotated based on their functions, domains, chemical properties, and structural proximity (see Supplementary Note).

### Hyper-mutated samples and double mutations

First, we listed all non-hyper-mutated tumors that have at least one mutation on the 54 genes carrying at least one double mutation. Then, for each double mutation, we noted the total number of non-synonymous mutations on these tumors and labeled the double mutant tumors as Double and the remaining gene-mutant tumors as Single (Supplementary Data 2).

To test the null hypothesis that the double mutant tumors (Double) have a lower or equal mutation burden compared to the remaining gene-mutant tumors (Single), we applied a permutation test (*p* < 0.01) with 5000 iterations. We prepared a two-column table having the Double/Single group labels of the tumors in the first column and the total number of non-synonymous mutations in the second column for each double mutation. To compare the observed and expected mean mutation counts for the two tumor groups, we shuffled the group labels in the first column 5000 times by preserving the second column as is. Here, we set the test statistic for two groups as follows:

1 $$Test\,Statistic=\mu (Double)-\mu (Single)$$

where μ is the mean mutation count. We calculated the permuted test statistic at each iteration by shuffling the Double/Single labels. At the end of 5000 iterations, we counted the number of iterations where the permuted test statistic is greater than the original test statistic (N) and found the *p*-value by N/5000.

### Allelic configuration of double mutations

We exploited supplementary data files of the papers^29–31^ to check *cis/trans* status of double mutations for the matching samples.

### Mutational signature analysis

We used 96 mutation contexts deposited in COSMIC that the format of codons and putative substitutions is as follows: C_1_[C_2_ > C_2_^subs^]C_3_ where C_i_ is the nucleotide in the corresponding position for *i* = 1,2,3 and C_2_ > C_2_^subs^ indicates the wild type nucleotide C_2_ is substituted by C_2_^subs^.

We assumed double mutations are of the same context either they have the same base pairs in C_1_[C_2_ > C_2_^subs^]C_3_ at the same position or C_1_, C_2_, C_2_^subs^, and C_3_ are mapped to the opposite strand with the same ordering^72^.

### Cell line network construction

We obtained a list of cell lines with the double mutations from Cell Model Passports and their drug response information from CancerrxGene^38,73^. We also extracted information about drug targets and target pathways. We used two different approaches to select drugs for *PTEN*, *APC*, and *PIK3CA* double mutant cell lines: if a drug is in the gray zone (|*z*-score|≤2) in the single mutant cell lines but gives a significant drug response in a double mutant cell line (|*z*-score|>2). If there is a single mutant cell line that is sensitive (or resistant) to the drug but the dual mutant cell line gives an opposite response to the drug. (Drug response flips sensitive into resistant or resistant into sensitive between single and dual mutant cell lines).

For *EGFR* we selected drugs that give significant drug response either in the single or double mutant cell line. Then we formed networks connecting mutations to cell lines, cell lines to drugs, and drugs to their target pathways.

### Patient-derived xenograft analysis

We used the mutation profiles, transcriptomic data and drug responses of patient-derived xenografts in^37^. We determined xenografts harboring significant doublets. Then, we compared changes in tumor volumes of single and dual mutant xenografts for the untreated and drug-treated cases (single mutation is part of a significant dual mutation). We preferred to specify the time intervals in multiples of 5. When a given timepoint is not a multiple of 5, we used linear interpolation between two nearest numbers containing a multiple of 5 as follows:

2 $${{{{{\mathrm{Vo}}}}}}{{{{{{\mathrm{l}}}}}}}_{i}={{{{{\mathrm{Vo}}}}}}{{{{{{\mathrm{l}}}}}}}_{i-1}+\frac{{t}_{i}-{t}_{i-1}}{{t}_{i+1}-{t}_{i-1}}({{{{{\mathrm{Vo}}}}}}{{{{{{\mathrm{l}}}}}}}_{i+1}-{{{{{\mathrm{Vo}}}}}}{{{{{{\mathrm{l}}}}}}}_{i-1})$$

where *t*_*i*_ is a timepoint that is multiple of 5 between the given timepoints *t*_*i*−1_ and *t*_*i*+1_ and Vol_*i*_ is the volume (mm^3^) at timepoint *i*.

### Reporting summary

Further information on research design is available in the Nature Portfolio Reporting Summary linked to this article.

## Supplementary information

Supplementary Information

Description of Additional Supplementary Files

Supplementary Data 1

Supplementary Data 2

Supplementary Data 3

Supplementary Data 4

Reporting Summary

## Data Availability

The results shown here are in whole or part based upon data generated by the TCGA Research Network: https://www.cancer.gov/tcga. The authors would like to acknowledge the American Association for Cancer Research and its financial and material support in the development of the AACR Project GENIE registry, as well as members of the consortium for their commitment to data sharing. Interpretations are under the responsibility of the study authors. The cell line data underlying the results presented in the study are available from GDSC in https://www.cancerrxgene.org/downloads, Cell Model Passports in https://cellmodelpassports.sanger.ac.uk/downloads, and The Cancer Dependency Map project in https://depmap.org/portal/download/. The PDX data underlying the results presented in the study are available in Gao et al.^37^. Source data of the main figures are available in 10.6084/m9.figshare.21788192.v3^74^.

## Code availability

Codes are available at https://github.com/bengiruken/LatentDriverDiscovery^75^.
