# Supplementary material for: Pan-cancer clinical impact of latent drivers from double mutations
Source: Commun Biol. 2023 Feb 20;6:202. doi: 10.1038/s42003-023-04519-5 (PMC9941481; doi:10.1038/s42003-023-04519-5)
Supplement: Supplementary file 1 — Supplementary Information [file 42003_2023_4519_MOESM1_ESM.pdf]

Supplementary Note

Variant Allele Frequency of Mutation Constituents

We created a dataset of 2917 distinct tumor-mutation pairs and associated VAF values based on the VAF values of each double mutation component on 1308 double mutant tumors and 295 double mutation constituents. Using the first quartile (Q1=0.21), median (0.30), and third quartile (Q3=0.40) values, the data was divided into four groups, each covering 25% of the total data. The values in each group encompass the VAF values 0.125-0.21, 0.21-0.30, 0.30-0.40, and 0.40-0.95. Figure S1 shows the histogram of VAF values for the mutations forming doublets among the double mutant tumors.

Annotation of Double Mutations

To find out spatial closeness of same gene double mutations we use 3DHotspots[1] which identifies statistically significant mutations clustering in 3D protein structures. There are 943 clusters of 504 different genes. If two mutated residues that are containing a dual mutation belong to the same cluster, we consider this same gene dual mutation components are in close proximity. We used Interactome Insider to identify if the components of either same gene or different gene double are located in the same interface [2]. Besides the experimental data in PDB and predicted data in Interactome3D, it also contains the predicted interfaces with their in-house method. We used EnrichR to find the pathway annotation of the genes having co-occurring mutations[3] .

Alterations in Chemical Properties of amino acids

In order to classify alterations with respect to chemical classes of amino acids before and after mutations, we prepared a file containing unique rows as follows "patient barcode| gene | residue number | wil type amino acid | mutant amino acid". We excluded the cases where the final amino acid is a stop codon. The 9 categories we evaluated in our analysis are Polar-Hydrophobic, Charged-Polar, Hydrophobic-Hydrophobic, Hydrophobic-Polar, Hydrophobic-Charged, Polar-Charged, Polar-Polar, Charged-Hydrophobic, Charged-Charged.

In total 188 missense mutations from 863 samples were analyzed. Positions that are mutated to multiple amino acids are counted multiple times.

We collected wild type and mutant residue information for each double mutant tumor and the double mutation constituent it comprises, as well as the chemical classifications of these residues. Considering that some mutations contribute to more than one double mutation, we ensured that each tumor and mutation was recorded only once. We excluded the cases where the mutant residue is a stop codon. Among the double mutant tumors on TSGs and OGs, there were 795 and 1170 unique tumor and mutation records, respectively.

PIK3CA Stability Analysis via Dynamut Tool

The mechanisms of activation of PI3Kα by some of the driver mutations have been recently worked out [1,4,5]. Unsurprisingly, considering their diverse mechanisms of action no clear trend is observed in the calculated folding free energy (ΔΔG) upon double or single mutation with DynaMut [6] (Figure S11). If the components of double mutations act via distinct mechanisms, the additivity of their activation potential is high; otherwise the additivity is low as in the E545/E542

example where the mutations execute the same mechanism of action. Using the inactive state (PDB id: 4OVV) we calculated the folding free energy (ΔΔG) upon mutation using DynaMut [6] to assess the impact of single and double mutations on PIK3CA stability. Unsurprisingly, considering their diverse mechanism of action no clear trend is observed (Figure S11). For example, H1047R is a strong driver that promotes interaction with the membrane. It destabilization impact is minor (ΔΔG ≈ -0.5 kcal/mol). The impact of weak drivers R88Q and R93W is somewhat stronger (ΔΔG ≈ -1.5 kcal/mol and ΔΔG ≈ -1 kcal/mol, respectively). The effect of allosteric mutation D539R is also minor (ΔΔG ≈ -0.6 kcal/mol). Another strong driver E542K (ΔΔG ≈ 0.7 kcal/mol), stabilizes the protein like the weak drivers D350G (ΔΔG ≈ 0.5 kcal/mol) and E453Q (ΔΔG ≈ 0.3 kcal/mol). The most prominent stability changes occur when the strong driver H1047R cooperates with the allosteric mutation P539R (ΔΔG ≈ -2.3 kcal/mol) and the minor mutation P104L (ΔΔG ≈ -2.5 kcal/mol). These two dual mutations H1047R/P539R and H1047R/P104L destabilize the protein as do T1025A/R88Q (ΔΔG ≈ 0.7 kcal/mol) while T1025A and R88Q have a destabilizing effect.

Mutational Signatures

For example, E542/E726 and E542/E545 are of context T[G>A]A in 20 and 10 records. Similarly, the context T[G>A]A forms the doublets E545/E726 and E545/M1004 in 20 and 4 records, respectively. E542 and E545 are strong driver mutations, while E726 and M1004 are strong and weak latent driver mutations. C[A>G]T forms H1047/H1048 doublet in 5 records, and C[G>A]A forms R88/R357 doublet in 7 records. Further examples include S97/L25 doublet in PIM1 with the context G[C>G]T (3 records) and S431/R232 doublet in PTPRD with the context T[C>T]G (3 records) (Supplementary Table 3).

Supplementary figures

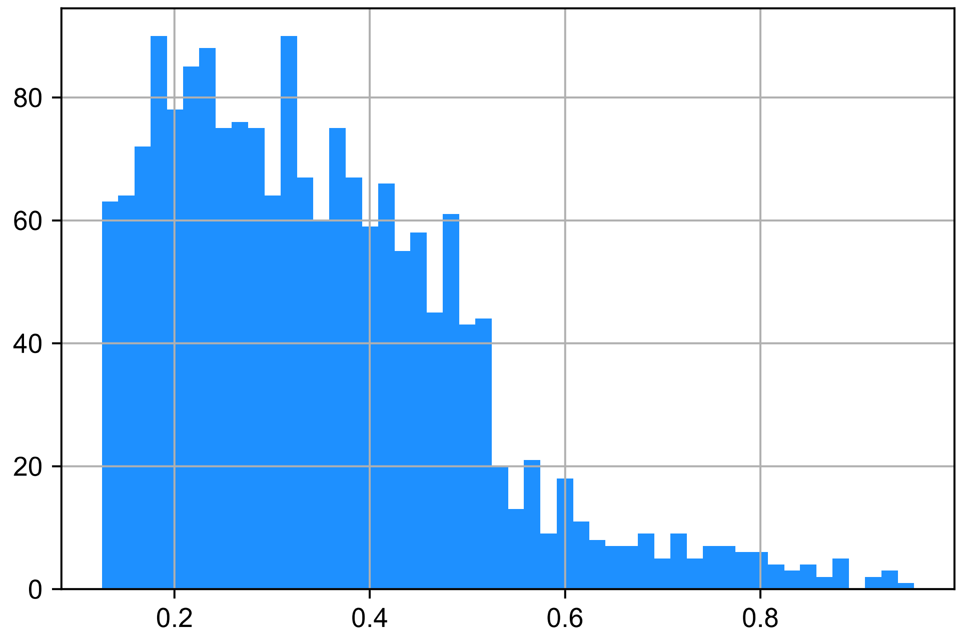

Figure S1: Histogram depicting VAF values of 213 double mutation constituents on 821 double mutant tumors. Double mutation constituents have VAF values accumulated between 0.2 and 0.4 on the double mutant tumors.

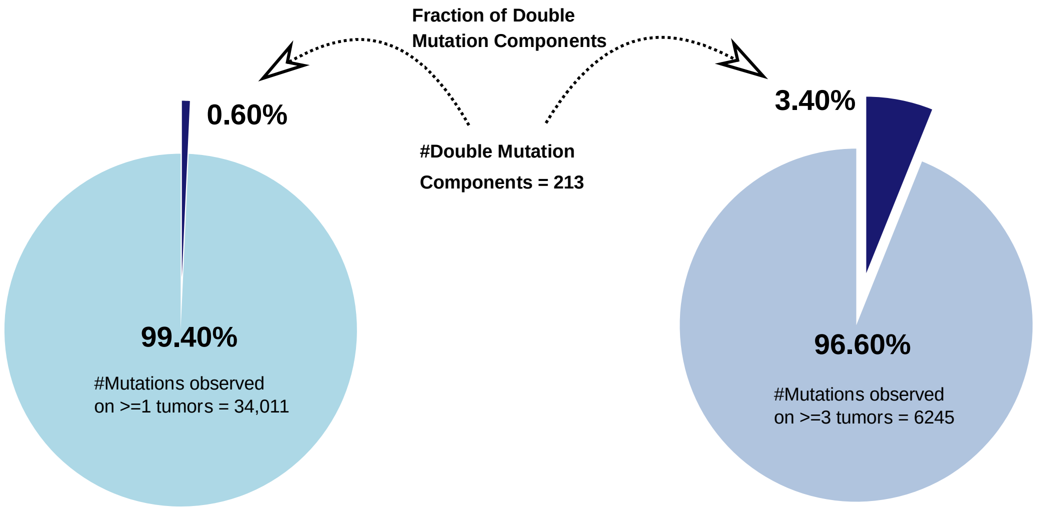

Figure S2: Fraction of 213 double mutation components among all mutations that are observed on at least one tumor (n=34,011)(on the left), and three tumors (n=6245)(on the right) on the 53 genes harboring at least one double mutation.

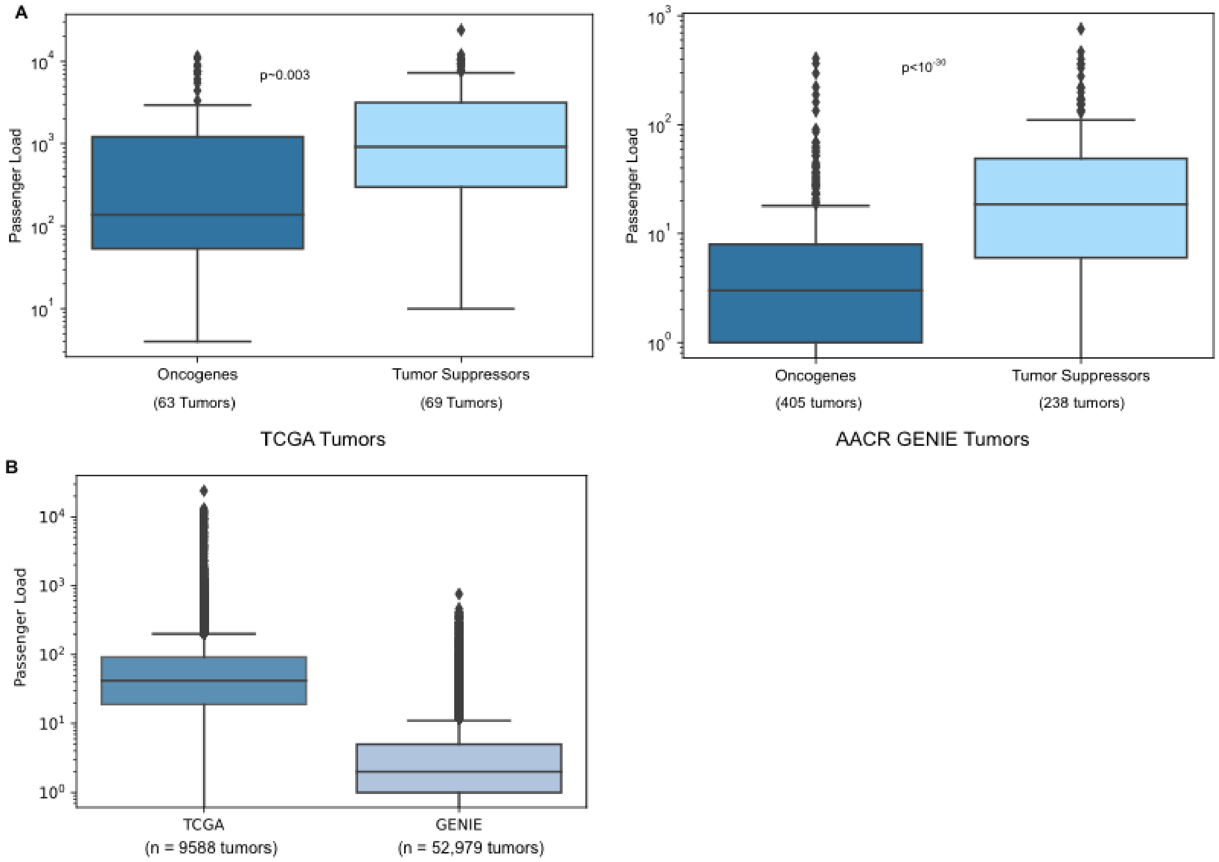

Figure S3: (A) Comparison of the passenger mutation loads of tumors from TCGA and GENIE data sets separately shows that the passenger load of the tumors carrying at least one tumor suppressor doublet is significantly higher than its counterpart in oncogenes. **(B)** Passenger mutation loads of the tumors among TCGA and GENIE data.

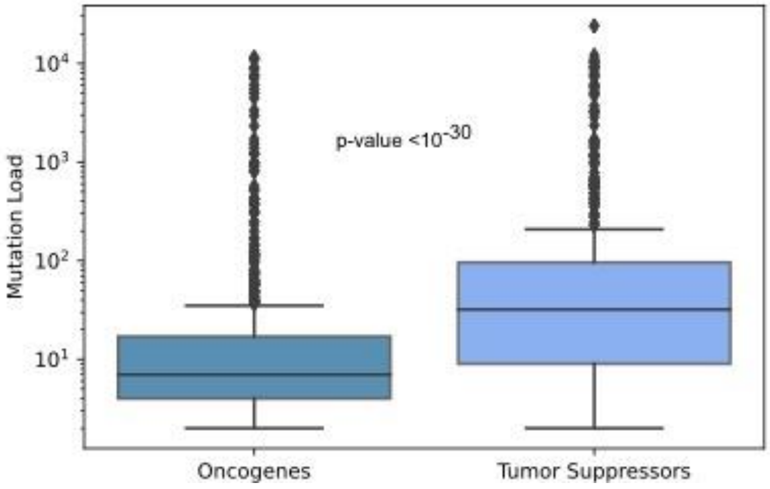

Figure S4: Mutation loads of tumors that that carry at least one significant double mutation on oncogenes (n=468) and tumor suppressor genes (n=307). There are 13 oncogenes and 25 tumor suppressor genes. Mutation loads of tumors with at least one double mutation on a TSG is siginificantly higher (Mann Whitney-U Test, $p<4x10^{-30}$).

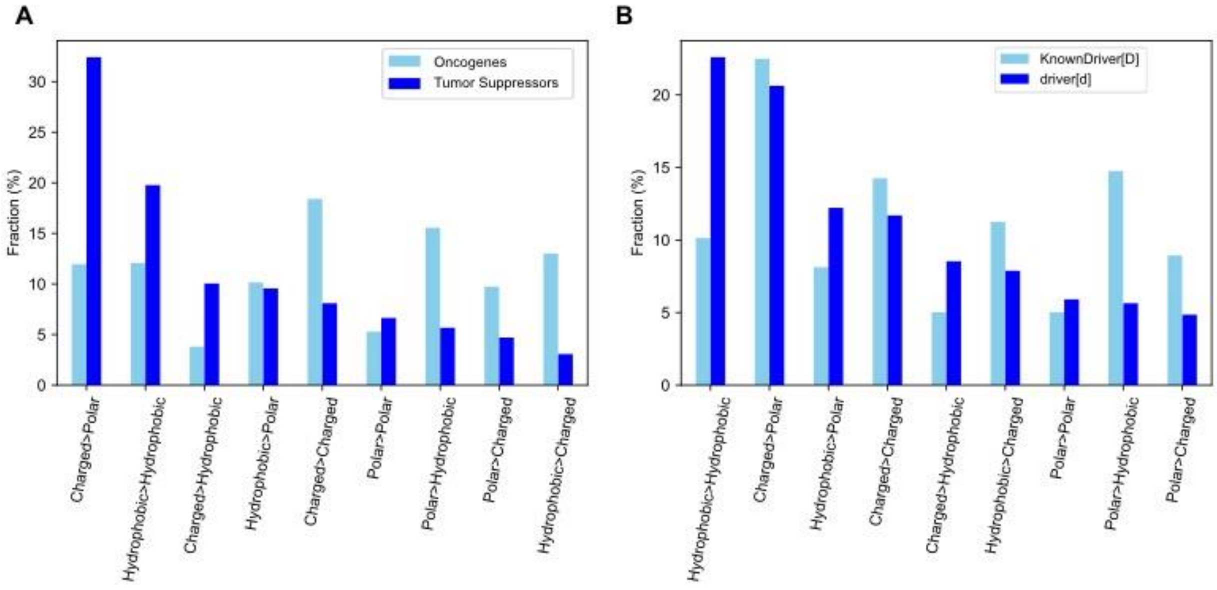

Figure S5: Analysis of the chemical class of (A) TSGs and OGs (B) known driver and latent driver mutations. Charged>Polar and Charged>Charged switches are more dominant among TSGs and OGs, Charged>Polar and Hydrophobic>Hydrophobic switches are more dominant among known driver and latent driver mutations, respectively.

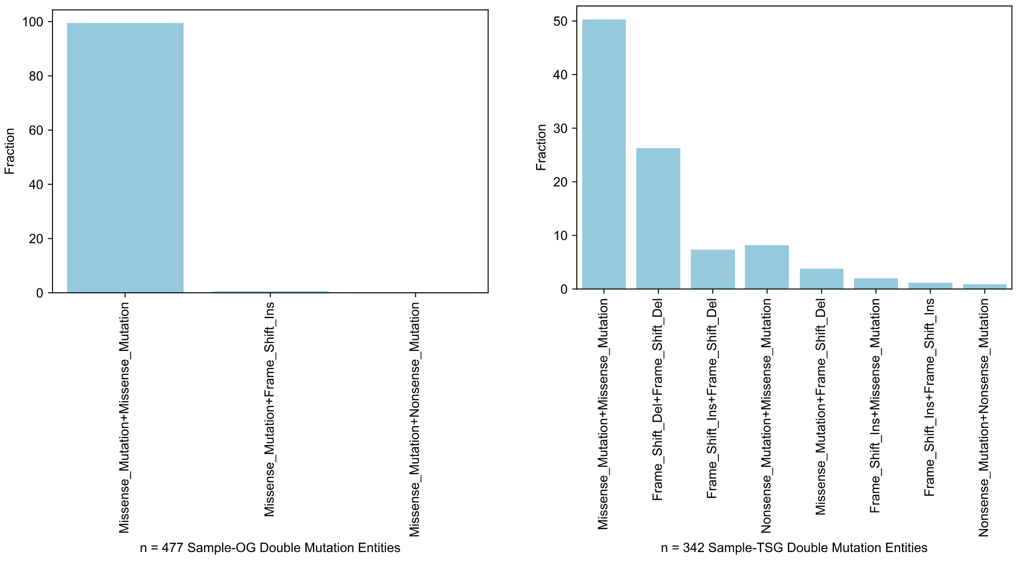

Figure S6: Combinations of missense mutations are highly prominent among double mutations on oncogenes; but for the doublets on tumor suppressor genes there are various combinations where missense+missense and frameshift_del +frameshift_del get the highest shares when the fractions (%) compared.

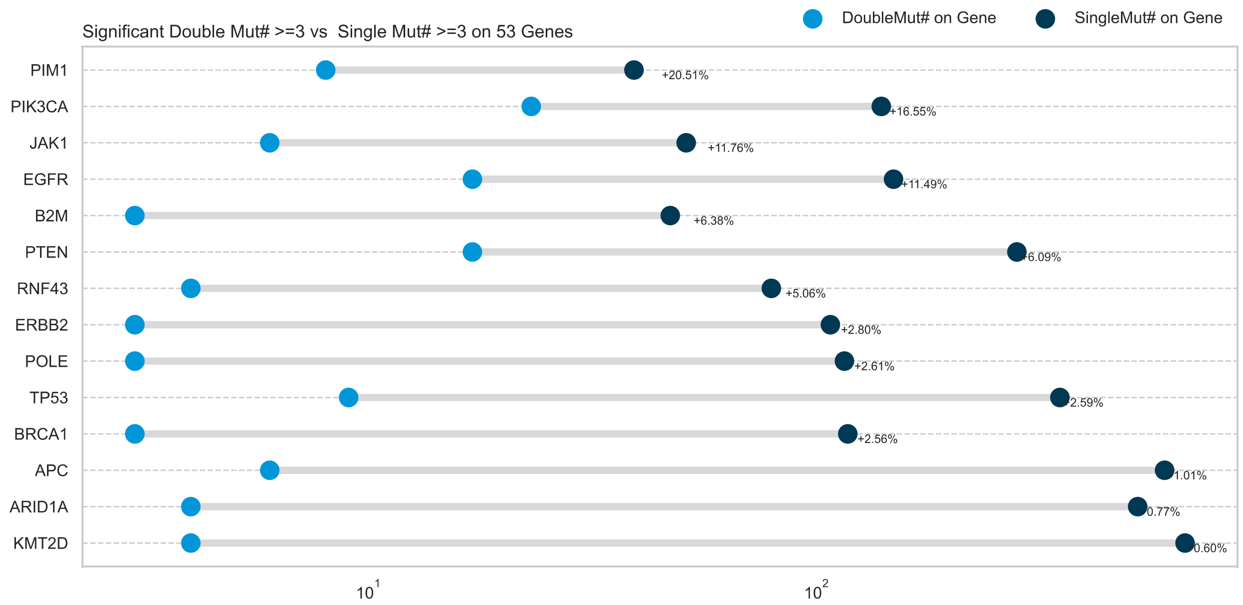

Figure S7: Number of double and single mutations on each gene that harbors at least one significant double mutation present in at least three double mutant tumors. All the single mutations observed on at least three tumors are included. Double mutation fraction among the single mutations are also noted.

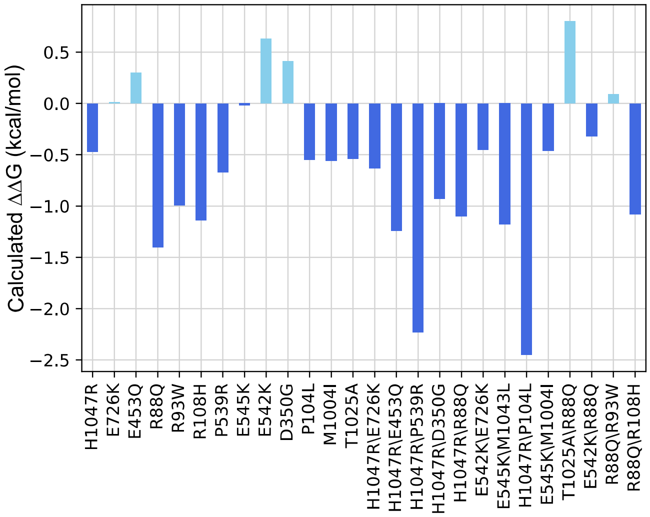

Figure S8: Predicted ΔΔG values for single and double mutations of PIK3CA calculated with Dynamut web server (PDB id: 4OVV).

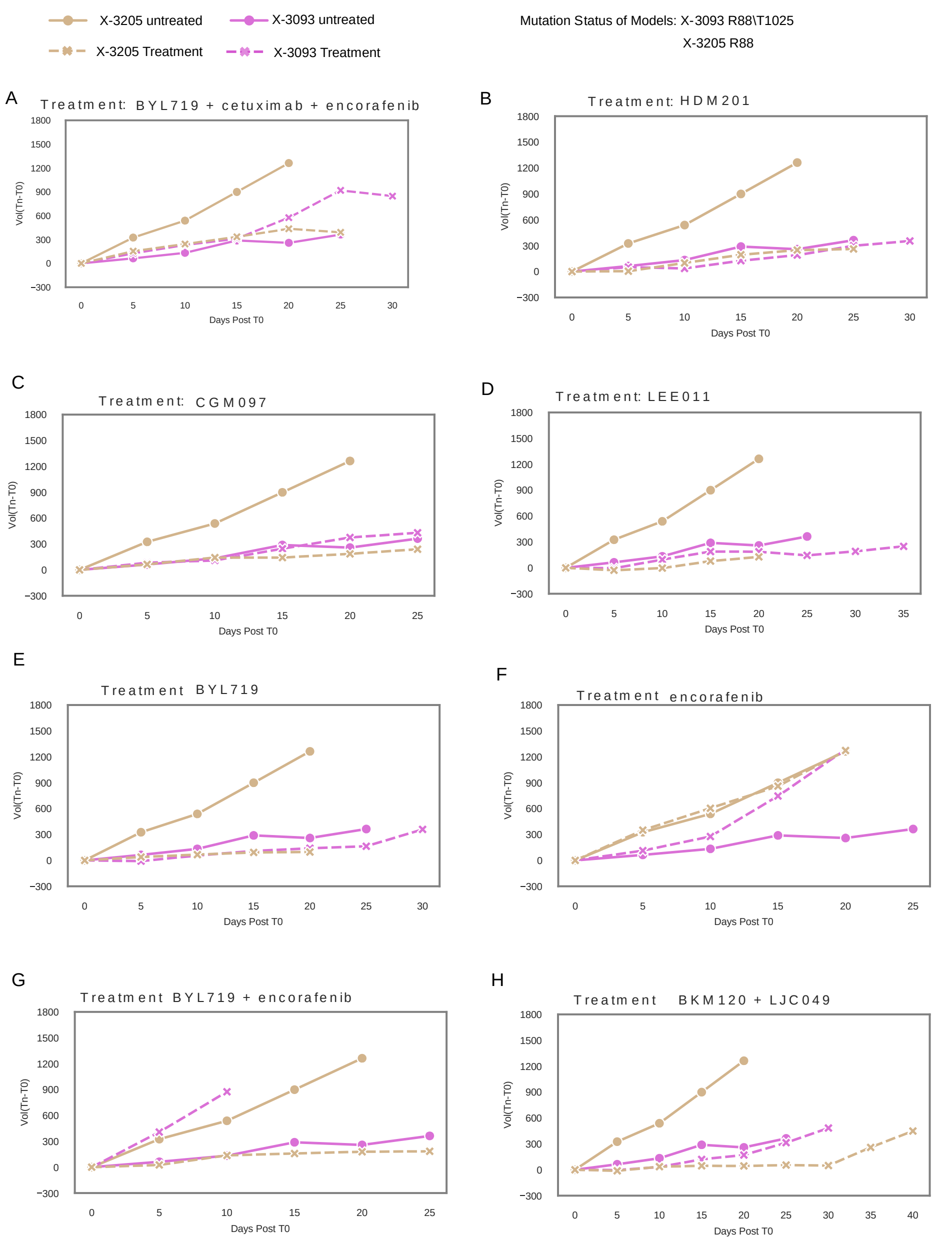

Figure S9. PIK3CA R88/T1025 mutant xenograft (X-3093, BRCA) volume change compared to single R88 mutant xenograft (X-3205, BRCA) for different drug treatments. x-axis shows treatment days, y-axis shows volume difference Volume(Day=n)-Volume(Day=0). Treatment with the drugs/drug combinations. **(A)** BYL719+cetuximab+encorafenib combination. **(B)**

HDM201 (Siremadlin). **(C)** CGM097. **(D)**LEE011(**Ribociclib**) **(E)** BYL719 (Alpelisib) **(F)** Encorafenib **(G)** BYL719+Encorafenib

(H) BKM120+LJC049

Supplementary References

1. Chen, S., He, X., Li, R., Duan, X. & Niu, B. HotSpot3D web server: an integrated resource for mutation analysis in protein 3D structures. *Bioinformatics* **36**, 3944–3946 (2020).

2. Meyer, M. J. *et al.* Interactome INSIDER: a structural interactome browser for genomic studies. *Nat. Methods* **15**, 107–114 (2018).

3. Kuleshov, M. V. *et al.* Enrichr: a comprehensive gene set enrichment analysis web server

2016 update. *Nucleic Acids Res.* **44**, W90–W97 (2016).

4. Zhang, M., Jang, H. & Nussinov, R. PI3K Driver Mutations: A Biophysical Membrane-Centric Perspective. *Cancer Res.* **81**, 237–247 (2021).

5. Zhang, M., Jang, H. & Nussinov, R. Structural Features that Distinguish Inactive and Active PI3K Lipid Kinases. *J. Mol. Biol.* **432**, 5849–5859 (2020).

6. Rodrigues, C. H. M., Pires, D. E. V. & Ascher, D. B. DynaMut: predicting the impact of mutations on protein conformation, flexibility and stability. *Nucleic Acids Res.* **46**, W350–W355 (2018).
