# Supplementary material for: Pan-cancer clinical impact of latent drivers from double mutations
Source: Commun Biol. 2023 Feb 20;6:202. doi: 10.1038/s42003-023-04519-5 (PMC9941481; doi:10.1038/s42003-023-04519-5)
Supplement: Supplementary file 2 — Description of Additional Supplementary Files [file 42003_2023_4519_MOESM2_ESM.pdf]

Description of Additional Supplementary Files

File name: Supplementary Data 1
Description: Significant double mutations and statistics.

File name: Supplementary Data 2
Description: Permutation test for robustness to hypermutated samples.

File name: Supplementary Data 3
Description: Tumor specific mutation channels of double mutation constituents.

File name: Supplementary Data 4
Description: Multiple corrections result.
